# Supplementary material for: Tracking Reservoirs of Antimicrobial Resistance Genes in a Complex Microbial Community Using Metagenomic Hi-C: The Case of Bovine Digital Dermatitis
Source: Antibiotics (Basel). 2021 Feb 23;10(2):221. doi: 10.3390/antibiotics10020221 (PMC7926801; doi:10.3390/antibiotics10020221)
Supplement: Supplementary file 1 [file antibiotics-10-00221-s001.pdf]

antibiotics

MDPI

Table S1. Forty bacterial taxa with ≥ 80% complete genomes detected in digital dermatitis lesions in cattle.

Cluster-ID	Top Reference	Complete (%)	Redundancy Score (%)	Abundance (%)	Contig N50	Genome Size	Contig Number	GC (%)
Cluster 1	*f_Spirochaetaceae*	100.00	98.01	0.30	27,479	2,336,932	146	39.28
Cluster 2	*f_Spirochaetaceae*	100.00	98.81	0.32	13,773	2,564,437	392	35.78
Cluster 3	p_*Bacteroidetes*	97.37	98.37	0.43	42,243	2,167,245	88	53.84
Cluster 4	*Porphyromonas_somerae*_DSM_23386	97.24	24.54	0.36	10,588	1,869,682	262	47.61
Cluster 5	*f_Spirochaetaceae*	96.95	98.77	0.21	7,022	2,422,999	486	41.41
Cluster 6	p_*Bacteroidetes*	96.90	98.36	0.70	33,542	2,244,038	95	51.02
Cluster 7	*Treponema_phagedenis*_4A	96.77	0.01	1.00	27,414	2,746,354	154	40.11
Cluster 8	c_*Betaproteobacteria*	96.30	94.31	0.26	24,410	2,003,539	116	43.85
Cluster 9	p_*Bacteroidetes*	96.16	99.58	0.88	7,345	2,957,833	688	53.78
Cluster 10	*f_Spirochaetaceae*	95.97	97.93	0.87	10,237	2,675,373	389	36.94
Cluster 11	*Treponema_medium*_ATCC_700293	95.36	19.24	0.51	22,682	2,649,495	189	44.11
Cluster 12	o_*Clostridiales*	95.34	98.75	0.30	8,208	1,457,254	278	38.38
Cluster 13	p_*Bacteroidetes*	95.08	99.16	0.78	29,233	2,286,428	120	53.86
Cluster 14	k_*Bacteria*	95.02	99.58	0.20	7,684	1,740,732	298	40.38
Cluster 15	*f_Spirochaetaceae*	93.74	82.51	0.87	11,054	2,858,440	430	37.30
Cluster 16	p_*Bacteroidetes*	93.50	98.72	0.20	6,679	2,261,481	488	52.61
Cluster 17	o_*Clostridiales*	92.63	98.28	0.21	6,493	2,200,433	488	36.93
Cluster 18	*f_Spirochaetaceae*	92.34	97.43	0.49	30,627	2,454,273	137	42.94
Cluster 19	*Porphyromonas_levii*_DSM_23370	92.32	1.32	1.04	12,522	1,969,961	226	46.16
Cluster 20	f_*Porphyromonadaceae*	92.32	99.14	0.36	9,297	1,556,924	223	41.84
Cluster 21	o_*Clostridiales*	91.61	96.55	0.26	17,698	1,595,341	151	37.99
Cluster 22	o_*Lactobacillales*	91.34	98.26	0.88	9,424	2,270,006	333	37.36
Cluster 23	*Streptococcus_henryi*_DSM_19005	90.18	13.13	0.25	12,445	2,261,995	355	38.92
Cluster 24	g_*Mycoplasma*	89.35	99.11	0.21	4,281	663,291	198	29.48
Cluster 25	k_*Bacteria*	88.12	89.54	1.21	7,326	1,820,058	350	43.13
Cluster 26	f_*Porphyromonadaceae*	87.35	97.73	0.26	6,262	1,634,006	318	44.96
Cluster 27	*Mycoplasma_fermentans*_JER	86.77	20.90	0.63	13,874	853,097	74	27.29

Cluster 28	f_*Spirochaetaceae*	86.64	99.54	0.30	27,998	1,801,454	87	33.25
Cluster 29	k_*Bacteria*	86.00	98.61	0.61	46,373	817,356	23	32.28
Cluster 30	c_*Gammaproteobacteria*	85.86	98.15	0.19	3,182	2,253,158	853	55.11
Cluster 31	o_*Clostridiales*	84.53	98.59	0.28	15,892	1,050,181	76	47.90
Cluster 32	k_*Bacteria*	84.45	99.53	0.19	3,643	1,232,133	425	37.31
Cluster 33	o_*Clostridiales*	84.35	98.12	0.31	10,189	1,268,755	206	34.29
Cluster 34	o_*Lactobacillales*	84.13	99.05	0.27	13,461	1,053,503	98	43.07
Cluster 35	o_*Clostridiales*	82.85	98.56	0.59	29,555	1,062,224	65	30.36
Cluster 36	k_*Bacteria*	81.72	98.54	0.19	4,452	1,667,328	472	29.57
Cluster 37	f_*Porphyromonadaceae*	81.53	98.54	0.26	26,381	1,410,665	63	51.56
Cluster 38	k_*Bacteria*	80.67	99.01	0.97	16,810	873,511	71	28.18
Cluster 39	o_*Clostridiales*	80.41	99.01	0.19	6,472	1,505,306	316	34.25
Cluster 40	o_*Clostridiales*	80.31	98.51	0.29	38,172	1,336,110	34	36.51

Table S2. Tetracyline resistance genes distribution in 40 bacterial taxa with ≥ 80% complete genomes detected in digital dermatitis lesions in cattle.

**Cluster-ID**	**Efflux Pump**						**Ribosomal Protection Proteins**									**Inactivation Enzyme**
	***tet*31**	***tet*33**	***tet*B**	***tet*H**	***tet*L**	***tet*Z**	***tet*32**	***tet*36**	***tet*40**	***tet*M**	***tet*O**	***tet*Q**	***tet*S**	***tet*T**	***tet*W**	***tet*X**
Cluster 1	0	0	0	0	0	0	0	0	0	7	0	0	0	0	0	0
Cluster 2	0	0	0	0	0	0	0	0	0	0	0	0	0	1	0	0
Cluster 3	0	0	0	0	0	0	0	0	0	0	1	44	0	0	0	0
Cluster 4	0	0	0	0	0	0	0	0	0	2	0	19	0	0	0	0
Cluster 5	0	0	0	0	0	0	0	0	0	0	0	0	0	0	0	0
Cluster 6	0	0	0	0	0	0	0	0	0	0	0	46	0	0	0	0
Cluster 7	0	0	0	0	0	0	0	0	0	2	63	1	0	0	0	0
Cluster 8	2	0	0	0	0	0	0	0	0	0	0	0	0	0	0	0
Cluster 9	0	0	0	0	0	0	0	0	0	0	0	22	0	0	0	0
Cluster 10	0	0	0	0	0	0	1	0	0	0	1	0	0	0	0	0
Cluster 11	0	0	0	0	0	0	0	0	0	14	0	0	0	0	0	0
Cluster 12	0	0	0	0	0	0	0	0	0	0	0	1	0	0	0	0
Cluster 13	0	0	0	0	0	1	0	0	0	0	1	121	0	0	1	0
Cluster 14	0	0	0	0	0	0	0	0	8	0	0	2	0	0	51	0
Cluster 15	0	0	0	0	0	0	0	0	0	0	0	2	0	0	0	0
Cluster 16	0	0	0	0	0	0	0	0	0	0	0	29	0	0	0	0
Cluster 17	0	0	0	0	0	0	30	0	0	0	1	1	0	0	6	0
Cluster 18	0	0	0	0	0	0	0	0	0	0	0	2	0	0	0	0
Cluster 19	0	0	0	0	0	0	0	0	0	0	0	86	0	0	1	0
Cluster 20	0	0	0	0	0	0	0	0	0	1	0	34	0	0	0	0

Table 2. continuation.

**Clus-ter-ID**	**Efflux Pump**						**Ribosomal Protection Proteins**									**Inacti-vation En-zyme**
	***tet*31**	***tet*33**	***tet*B**	***tet*H**	***tet*L**	***tet*Z**	***tet*32**	***tet*36**	***tet*40**	***tet*M**	***tet*O**	***tet*Q**	***tet*S**	***tet*T**	***tet*W**	***tet*X**
Cluster 21	0	0	0	0	0	0	2	0	3	0	14	9	0	0	11	1
Cluster 22	0	0	0	0	0	0	0	0	0	1	0	0	0	0	0	0
Cluster 23	0	0	0	0	0	0	0	0	0	1	0	0	0	0	1	0
Cluster 24	0	0	0	0	0	0	0	0	0	0	0	0	0	0	0	0
Cluster 25	0	1	0	0	0	0	0	0	0	1	1	136	0	0	0	0
Cluster 26	0	0	0	0	0	0	0	0	0	0	1	19	0	0	0	0
Cluster 27	0	0	0	0	0	0	0	0	0	1	0	0	0	0	0	0
Cluster 28	0	0	0	0	0	0	0	0	0	0	0	0	0	0	0	0
Cluster 29	0	0	0	0	0	0	0	0	0	0	0	0	0	0	0	0
Cluster 30	1	0	0	0	0	0	0	0	0	0	0	6	0	0	1	0
Cluster 31	0	0	0	0	0	0	0	0	4	0	8	0	0	0	0	0
Cluster 32	0	0	0	0	0	0	0	0	0	0	0	0	0	0	0	0
Cluster 33	0	0	0	0	0	0	0	0	0	0	0	0	0	0	0	0
Cluster 34	0	0	0	0	0	0	0	0	0	0	0	0	0	0	0	0
Cluster 35	0	0	0	0	0	0	0	0	0	2	47	1	0	0	0	0
Cluster 36	0	0	0	0	0	0	0	0	0	0	0	0	0	0	0	0
Cluster 37	0	0	0	0	0	0	0	0	1	0	0	15	0	0	0	0
Cluster 38	0	0	0	0	0	0	0	0	0	1	0	1	0	0	0	0
Cluster 39	0	0	0	0	0	0	0	0	0	0	0	0	0	0	0	0
Cluster 40	0	0	0	0	0	0	0	0	0	0	1	0	0	0	2	0
